# Evaluation of Artesunate and Praziquantel Combination Therapy in Murine *Schistosomiasis mansoni*

**Published:** 2018

**Authors:** Laila Abdel Moniem HEGAZY, Mona Hussein Al MOTIAM, Naglaa Fathy ABD EL-AAL, Shereen Mahmoud IBRAHIM, Heba Khalil MOHAMED

**Affiliations:** 1. Dept. of Medical Parasitology, Faculty of Medicine, Zagazig University, Zagazig, Egypt; 2. Dept. of Pathology, Theodor Bilharz Research Institute (TBRI), Giza, Egypt

**Keywords:** *Schistosoma mansoni*, Artesunate, Praziquantel, Apoptosis, P53, Bcl-2

## Abstract

**Background:**

Despite the global efforts to control schistosomiasis, still prevalence in endemic regions unchanged. The present study was conducted to investigate the possible role of artesunate (AS) and praziquantel (PZQ) combination in enhancing cure in pre-patent and patent *Schistosoma mansoni* infection, and study the role of apoptosis in evaluation of the drugs efficacy.

**Methods:**

Eighty laboratory-bred Swiss albino male mice were classified into four groups (20 mice each); control, PZQ treated (500 mg/kg), AS treated (400 mg/kg) and combined AS (400 mg/kg) + PZQ (500 mg/g) groups. Efficacy of the drugs was assessed by parasitological (egg count/gram stool, worm burden, tissue egg load, oogram pattern), histopathological (haematoxylin and eosin –for detection of type of hepatic granulomas, number & diameter) and immunohistochemical studies (P53 and Bcl-2 markers for determination of inflammatory cells and the degree of apoptosis).

**Results:**

Significant reduction was recorded in stool egg count, tissue egg count (liver and intestine), worm burden, granuloma number and size and changed oogram patterns in artesunate -praziquantel combined group followed by artesunate monotherapy group. There was a significant increase in the apoptotic proteins P53 and slight increase in anti-apoptotic proteins Bcl-2 in the infected group compared to the control healthy group. A significant decrease and increase in P53 & Bcl-2 expressions respectively were observed in artesunate – praziquantel combined group compared to control infected group.

**Conclusion:**

artesunate-praziquantel combination is a potential upcoming chemotherapy for schistosomiasis *mansoni*. Both Bcl-2 and P53 are good markers assessing *S. mansoni* apoptosis, morbidity and chemotherapy efficacy.

## Introduction

Schistosomiasis is a major public health problem in many developing countries. More than 258 million people are infected by different *Schistosoma* species, about 700 million persons are at risk of infection in 78 countries where schistosomiasis is endemic with moderate to high transmission, that need repeated preventive treatment over a number of years to reduce and prevent morbidity (1).

Praziquantel (PZQ) is considered the main drug for schistosomiasis morbidity control, its low cost, safety and efficacy against adult worms of all schistosome species led to its widespread use. Despite two decades of widespread treatment of schistosomiasis with PZQ, the number of infected patients with schistosomiasis is still high (2).

Moreover, long-term use of PZQ, owing to the strong dependence on the drug and its repeated use may induce drug resistance or decreased susceptibility, appearance of such PZQ-resistant strains may put the drug out of use (3).

Since, PZQ fails to prevent re-infection and has minor effect on immature worms, which can result in low cure rates in areas where schistosomiasis is hyperendemic (4). Thus, there is an ongoing need to develop alternative drugs to overcome schistosomiasis (5).

Adult schistosomes were similar to Plasmodium protozoa, causing malaria, in feeding on blood and hemoglobin degradation into hemozoin. The interference with hemozoin development in *S. mansoni* is an important mechanism of schistosomicidal action of the anti-malarial compounds and highlights the haem crystallization development as an effective therapeutic goal to treat the disease (6).

Artesunate, one of artemisinin group an anti-malarial treatment, was a promising drug for treatment and chemoprophylaxis of schistosomiasis (7). Combination therapy of artesunate and PZQ resulted in complete disappearance of tissue eggs as well as 100% reduction rate in total worm count (8).

Pathologic apoptosis is an unregulated process and can be massive that leads to cell lysis, inflammatory response, and serious health problems; this is unlike physiological apoptosis which is regulated carefully and does not involve any secondary events (9). P53 tumor suppressor protein; acts as a protector of genomic activity by induction of either cell cycle arrest or programmed cell death (apoptosis) (10).

Bc1-2 (B cell lymphoma); has a significant role in regulating cell survival and apoptosis (11). Bcl-2 gene is an anti-apoptotic protein; participate in the p53 apoptotic pathway and the equilibrium between those proteins is essential for the susceptibility to apoptosis (12). Previous studies relied on immunohistochemical studies for detection of immunoreactivities of Bcl-2 (13) and P53 as apoptotic markers for evaluation of the therapeutic effect of antischistosomal drugs (12).

PZQ and artesunate display broad-spectrum antischistosomal activities by two different mechanisms, and target different developmental parasite stages, the current study was conducted to assess the possible role of artesunate- praziquantel combination in enhancing cure in pre-patent and patent *Schistosoma mansoni* infection, and study if a similar or comparable efficacy might be achieved using their monotherapy. The assessment was depending on parasitological, histopathological studies, and immunohistochemical expression of P53 and Bcl-2 in hepatocyte induced apoptosis.

## Materials and Methods

This nonrandomized control-trial study was performed at Parasitology Department, Faculty of Medicine, Zagazig University and Theodor Bilharz Research Institute (TBRI), Giza, Egypt from May 2014 to Jun 2015.

### Experimental Mice

Eighty laboratory-bred, parasite free Swiss albino male mice, 4–6 wk old, weighing 20–25 gm each, were obtained from Schistosome Biological Supply Center at TBRI. The mice were maintained on a standard commercial stock pelleted diet with free accessible water and kept under fixed appropriate conditions of housing and handling in an air-conditioned animal house at 20–22 °C all over the time of this study.

### Mice infection

The mice were prepared and infected in TBRI using ±100 *S. mansoni* cercariae obtained from infected *Biomphalaria alexandrina* snails by subcutaneous injection method (14), and control healthy group was considered.

### Ethics statement

Experimental mice were used according to international guidelines approved by the Ethics Committee for Animal Experimentation, Faculty of Medicine, Zagazig University, Egypt.

### Drugs

Praziquantel (PZQ) used was distocide (EPICO, Egypt). The drug was given in the form of aqueous suspension in a solution of 3% ethanol and 7% Tween 80 at a concentration of 40 g/L (500mg/kg). Artesunate (AS) Sigma-Aldrich was suspended in a solution of 3% ethanol and 7% Tween 80 and water just before treatment (400 mg/kg). Both drugs were administered orally to each mouse by gavage through mouth (15).

### Experimental design and treatment protocol

The mice were classified into four groups (20 mice each) and each group subdivided into 2 subgroups (10 mice each): I-Control group (C): Control healthy (CH) 10 mice uninfected (normal); Control infected (CI) 10 mice infected untreated. II-Praziquantel (PZQ) group: PZQ3 10 mice infected then treated with praziquantel monotherapy (500 mg/kg) single oral dose 3 wk (w) post infection (pi); PZQ6: 10 mice infected then treated with praziquantel monotherapy (500 mg/kg) single oral dose 6w pi. III-Artesunate (AS) group: AS3 10 mice infected then treated with artesunate monotherapy (400 mg/kg) single oral dose 3w pi; AS6 10 mice infected then treated by artesunate monotherapy (400 mg/kg) single oral dose 6w pi. IV-Combined (AS+ PZQ) group: AS+ PZQ3 10 mice infected then treated by artesunate (400 mg/kg) and praziquantel (500 mg/kg) single oral dose combination therapy 3w pi; AS+ PZQ6 10 mice infected then treated by artesunate (400 mg/kg) and praziquantel (500 mg/kg) single oral dose combination therapy 6w pi.

### Assessment of therapeutic effect

Eight weeks post infection all mice groups were sacrificed using rapid decapitation under anesthesia (16) for assessment of drug efficacy by:

#### 1. Parasitological parameters

A: Egg count per gram stool was performed according to modified Kato-thick smear technique (17), started from 6^th^-week pi to ensure infection and assess the effect of drug therapy. B: Worm burden (WB): Worms recovered by liver and intestinal perfusion (18). C: Tissue egg load: Calculated by number of eggs/gram tissues (liver and intestine) (19). The induced percentage reduction (R%) in worm burden and egg number was calculated (20) *P*=CV/Cx100 (Where P: percentage; C: mean number of WB or eggs recovered from control infected mice; V: mean number of WB or eggs recovered from infected treated mice). D: Oogram: The percentage of eggs at various developmental stages in the small intestine was determined (21).

#### 2. Histopathological study

Ten percent of Formalin-fixed paraffin-embedded livers of all studied groups were cut into 4 μm sections. Liver sections stained with Haematoxylin and Eosin (H&E). Sections were examined for periovular granulomas. The diameter of granulomas was measured using the ocular micrometer. The mean diameter for each group was calculated. Granuloma count was conducted in five successive fields of magnification from serial tissue sections more than 25 um apart (22).

#### 3. Immunohistochemical study

Paraffin-embedded liver sections were used for immunohistochemical study using the avidin biotin peroxidase method (23). These sections from all different studied groups were immunohistochemically stained by P53 and Bcl-2 using the Dako autostainer for determination of the extent of inflammatory cells and detection of the degree of apoptosis.

### Data analysis

Data were entered and analyzed using SPSS version 19 under Windows 7 (Chicago, IL, USA). Results were expressed as mean ± standard deviation (SD). The statistical analyses were done by Student’s t*-*test and oneway ANOVA test. *P*-value <0.001 was considered significant.

## Results

### Parasitological results

**Fig. 1:**
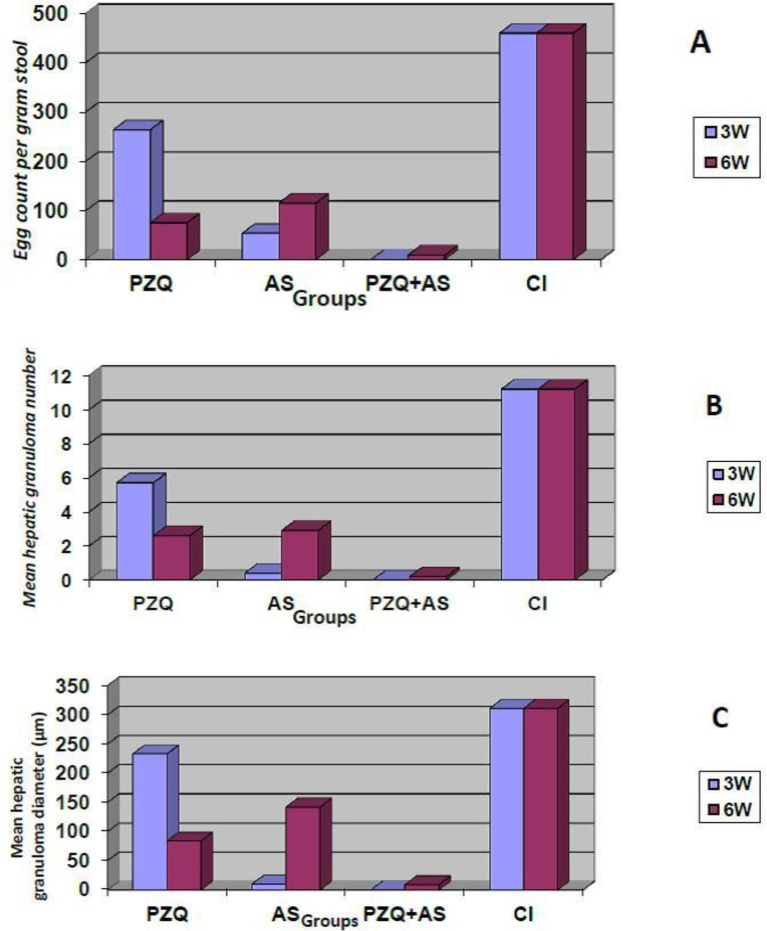
**A)** Mean egg count/gram stool in groups treated with PZQ, AS and their combination 2.6±(460.0±22.5), PZQ group (263.5±14.5& 99.0±19.1), AS group (53.5±11.8 & 115.0±14.3) and PZQ+AS group (0±0& 9.0±3.9); using one-way ANOVA test; PZQ+AS is the most effective group (p>0.001); CI (control infected), PZQ (praziquantel), AS (artesunate); pi (post infection). **B)** Mean hepatic granuloma number in groups treated with PZQ, AS and their combination (PZQ+AS) in *S. mansoni* infected mice; showed mean±SD 3&6 weeks pi respectively for CI (11.2±1.0); PZQ group (5.7±0.5& 2.6± 0.5); AS group (0.4±0.3& 2.9±0.9); PZQ+AS (0±0 & 0.2±0.4); using one-way ANOVA test; PZQ+AS is the most effective group (p>0.001). **C)** Mean hepatic granuloma number in groups treated with PZQ, AS and their combination (PZQ+AS) in *S. mansoni* infected mice; showed mean±SD 3&6 weeks pi respectively for CI (311.0±9.9); PZQ (233.0±12.5&84.0±11.7); AS (10.0±21.1& 142.0±13.2); PZQ+AS (0±0&9.0±22.3) using one-way ANOVA test; PZQ+AS is the most effective group (*P*>0.001)

Regarding the mean egg count/gram stool, the highest reduction in the mean egg count/gram stool was observed in AS+PZQ3 treated combined group followed by AS+PZQ6, AS3, PZQ6 then AS6 lastly PZQ3 groups with percent reduction 100%, 98%, 88.4%, 78.5% then 75% lastly 42.7% respectively compared to infected control group (Fig. 1A).

Regarding the worm burden, the highest reduction in the mean female and total worm burden were observed in AS+PZQ6 treated combined group followed by AS+PZQ3, PZQ6, AS3, AS6 Lastly PZQ3 groups with percent reduction (97%, 96%, 89%, 71%, 62 lastly 45%) and (95.4%, 94.9%, 87.2% 68.4 then 66.3% lastly 37.2%) in female and total worm burden respectively compared to their corresponding control infected group (Table 1).

**Table 1:** Effect of praziquantel, artesunate and their combination on male, female and total worm burden in *S. mansoni* infected mice 3 & 6 wk pi in all studied groups

***Groups***	***Mean number of worms ± SD***												
***Weeks pi***	**CI**			**PZQ**			**AS**			**AS+PZQ**		
	**Male**	**Female**	**Total**	**Male**	**Female**	**Total**	**Male**	**Female**	**Total**	**Male**	**Female**	**Total**
3W	9.4±3.5	10.2±3.5	19.6± 6.2	6.7±1.6^#^	5.6± 1.6^*#^	12.3±1.9^*#^	3.3±1.3^*^	2.9±1.3^*^	6.2±1.7^*#^	0.6±0.5^*^	0.4±0.5^*^	1.0±0.9^*^
%R				28.7%	45%	37.2%	65%	71.5%	68.4%	93.6%	96%	94.9%
6W				1.4±0.8^*^	1.1± 0.7^*^	2.5±0.7^*^	2.8±1.9^*#^	3.8±1.4^#^	6.6±1.4^*#^	0.6±0.5^*^	0.3±0.5^*^	0.9±0.7^*^
%R				85%	89%	87.2%	70%	62.7%	66.3%	93.6%	97%	95.4%

*P*<0.05

* Significant different from the corresponding infected control group;

# Significant different from the corresponding

PZQ+AS combined group; CI (control infected), PZQ (praziquantel), AS (artesunate); pi (post infection); %R= percentage reduction

Regarding tissue egg count, the highest reduction in the mean egg count/gram (liver & intestine) were observed in AS+PZQ3 combined group followed by AS+PZQ6, AS3, PZQ6 then AS6 lastly PZQ3 groups with percent reduction (100%, 99.2% 94.7%, 90.8%, 90.3% lastly 38.5%) and (100%, 99.1%, 95.3%, 94.7%, 91.2% lastly 36.9%) respectively for liver and intestinal egg count compared to their corresponding infected control group (Table 2).

**Table 2:** Effect of praziquantel, artesunate and their combination on the egg count/gram tissue (liver& intestine) in *S. mansoni* infected mice 3 & 6 wk pi in all studied groups

***Groups***	***Mean egg count/gram tissue (liver & intestine) ± SD***							
	**Mean egg count/gram liver**				**Mean egg count/gram intestine**			
***Weeks pi***	**CI**	**PZQ**	**AS**	**AS+PZQ**	**CI**	**PZQ**	**AS**	**AS+PZQ**
3W	5450.0±738.2	3350.0± 392.3^*#^	290.5± 32.2^*^	0.0± 0.0^*^	7150.0±914.4	4510.0±1041.8^*#^	334.0±32.7^*^	0.0±0.0^*^
%R		38.5%	94.7%	100%		36.9%	95.3%	100%
6W		499.0± 39.0^*#^	530.0± 27.1^*#^	44.0±17.1^*^		290.5± 32.2^*^	632.0±32.6^*#^	62.0±17.5^*^
%R		90.8%	90.3%	99.2%		94.7%	91.2%	99.1%

*P*<0.05

* Significant different from the corresponding infected control group;

# Significant different from the corresponding

PZQ+AS combined group; CI (control infected), PZQ (praziquantel), AS (artesunate); pi (post infection); %R= percentage reduction

**Fig. 2:**
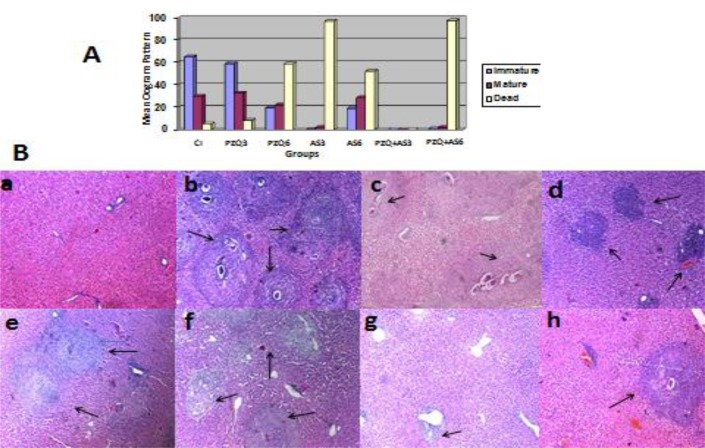
**A)** Oogram pattern in groups treated with PZQ, artesunate and their combination in *S. mansoni* infected mice 3 and 6 weeks post-infection; using one-way ANOVA test, PZQ+AS3 combined group showed highly significant different from all groups (P>0.001). **B)** Sections in the liver of *S. mansoni* control and treated mice groups (H&E X100) showing: **(a)** control healthy group with normal hepatic architecture; **(b)** infected control group with well circumscribed cellular granulomas with central fresh ova; **(c)** infected mice treated with PZQ 3w pi with multiple cellular granulomas with central ova; **(d)** infected mice treated with PZQ 6w pi with few granulomas with degenerated ova; **(e)** infected mice treated with AS 3w pi with a small cellular granuloma with central degenerated egg; **(f)** infected mice treated with AS 6w pi with cellular granulomas with degenerated central ova; **(g)** infected mice treated with PZQ+AS 3w pi with few inflammatory cells with no granulomas; **(h)** infected mice treated with PZQ+AS 6w pi with small fibrocellular granulomas with degenerated ova. CI (control infected), PZQ (praziquental), AS (artesunate)

Regarding the oogram, the highest mean dead egg count and lowest mean immature & mature egg count were observed in AS+PZQ6 combined group followed by AS+PZQ3, PZQ6, AS3 then PZQ3 groups compared to infected control group (Fig. 2A).

### Histopathological results

The highest reduction in the mean hepatic granuloma number & diameter were observed in PZQ+AS3 combined group followed by PZQ+AS6, AS3, PZQ6, AS6 lastly PZQ3 groups with percent reduction (100%, 98.2%, 96.4%, 76.8%, 74.1% lastly 49%) and (100%, 97.1%, 96.8% 73%, 54.3% lastly 25.1%) respectively for the mean granuloma number and diameter compared to their corresponding infected control group (Fig. 1B, C & Fig. 2B).

### Immunohistochemical results

**Fig. 3:**
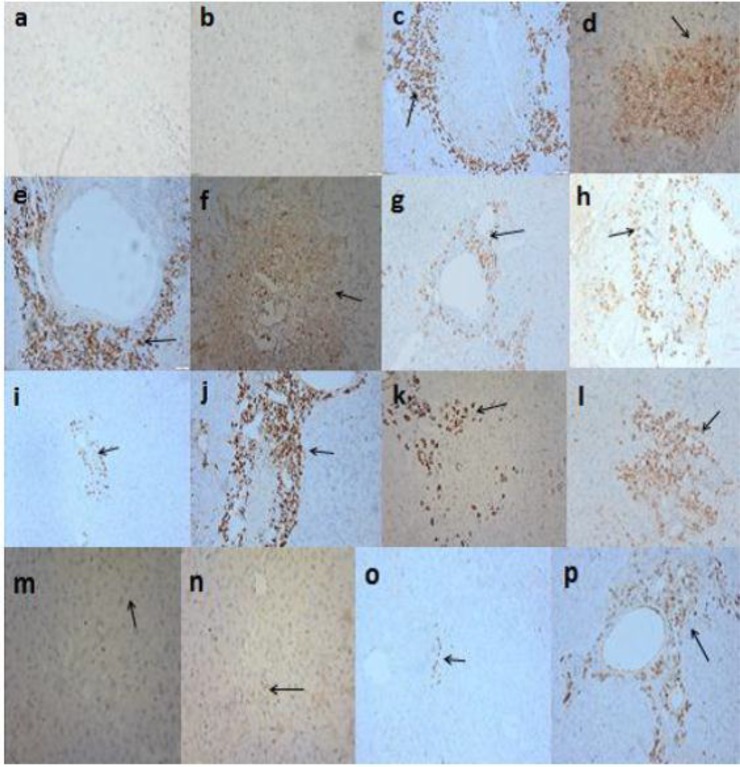
Sections in the liver of *S. mansoni* control and treated mice groups showing **(a)** control healthy mice group with negative reactions for P53, grade 0 (P53 Immunohistochemistry ×200); **(b)** control healthy mice group with negative reactions for Bcl-2, grade 0 (Bcl-2 Immunohistochemistry ×200); **(c)** infected control mice group with strong positive reactions for P53, grade 3 (P53 ×200); **(d)** infected control mice group with mild reactions for Bcl-2, grade 1 (Bcl-2 ×200); **(e)** infected mice group treated with PZQ 3w pi with strong reactions for P53, grade 3 (P53 ×200); **(f)** infected mice group treated with PZQ 3w pi with mild reactions for Bcl-2, grade 1(Bcl-2 ×200); **(g)** infected mice group treated with PZQ 6w pi with mild reactions for P53, grade 1(P53 ×200); **(h)** infected mice group treated with PZQ 6w pi with strong reactions for Bcl-2, grade 3 (Bcl-2 ×200); **(i)** infected mice group treated with AS 3w pi with a very mild reactions for P53 of grade 1 (P53 ×200); **(j)** infected mice group treated with AS 3w pi with strong positive reactions for Bcl-2, grade 3 (Bcl-2 ×200); **(k)** infected mice group treated with AS 6w pi with moderate reactions for P53, grade 2 (P53 ×200); **(l)** infected mice group treated with AS 6w pi with moderate reactions for Bcl-2, grade 2 (Bcl-2 ×200); **(m)** infected mice group treated with AS+PZQ 3w pi with very mild reactions for P53, grade 0 (P53 ×200); **(n)** infected mice group treated with AS+PZQ 3w pi with negative reactions for Bcl-2, grade 0 (Bcl-2 ×200); **(o)** infected mice group treated with AS+PZQ 6w pi with mild reactions for P53, grade 1 (P53 ×200); **(p)** infected mice group treated with AS+PZQ 6w pi with strong reactions for Bcl-2, grade 3 (Bcl-2 ×200)

The highest reduction in the mean percentage of P53 marker was observed in AS+PZQ3 combined group followed by AS+PZQ6, AS3, PZQ6 then AS6 lastly PZQ3 groups with percent reduction (98.8%, 97.9%, 85.8%. 83.9%, 69.5% lastly 69.1%) and the highest increase in the mean percentage of Bcl-2 marker was observed in AS+PZQ3 combined group followed by AS+PZQ6, AS3, AS6, then PZQ6 lastly PZQ3 groups with percent reduction (negative reaction, 134%, 107%, 96% then 89% lastly 42%) compared to their corresponding infected control groups (Table 3 and Fig. 3).

**Table 3:** Effect of praziquantel, artesunate and their combination on expression of P53 & Bcl-2 markers in *S. mansoni* infected mice 3 & 6 wk pi in all studied groups

*Groups*	*Mean percentage of Immunohistochemistry ±SD*									
	Mean percentage of P53±SD					Mean percentage of Bcl-2±SD				
**Weeks pi**	CH	CI	PZQ	AS	AS+PZQ	CH	CI	PZQ	AS	AS+PZQ
3W	0.3±0.7	24.3±4.0	7.4±2.2^*#^	3.5±1.0^*^	0.3±0.7^*^	0.0±0.0	4.5±1.2	6.4±2.2^*^	9.3±1.6^*#^	0.0±0.0^*^
%R			69.5%	85.8%	98.8%			42%	107%	-ve reaction
6W			3.9±1.0^*^	7.5±1.5^*#^	0.5 ±1.8^*^			8.5±1.5^*#^	8.8±1.4^*^	10.5±1.4^*#^
%R			83.9%	69.1%	97.9%			89%	96%	134%

*P*<0.05

* Significant different from the corresponding infected control group;

# Significant different from the corresponding

PZQ+AS combined group; CH (control healthy), CI (control infected), PZQ (praziquantel), AS (artesunate); pi (post infection); %R= percentage reduction

Scoring the changes in P53, Bcl-2 expressions in different studied groups was done depending on the extension of the dark, brownish precipitate appeared at the positive slides into 4 grades as follows: 0: negative reaction, was given to unstained sections, 1: mild reaction, 2: moderate reaction and 3: strong reaction (24). In this context, our results showed negative correlation between Bcl-2 and p53 immunohistochemical markers expression in all studied groups (Fig. 3).

## Discussion

Artesunate, antimalarial artemisinin derivatives, is new promising antischistosomal compounds with well-tolerated therapeutic dosage range and is also available as a drug in itself. Artesunate is recognized to be less toxic among these several artemisinin derivatives especially when given orally and safer because it is absorbed and eliminated rapidly (25).

In the current study, we demonstrated that the treatment with artesunate combined to PZQ exceeded PZQ monotherapy. Besides, artesunate monotherapy was nearly effective as the combined treated group regarding the reduction in the mean egg count/gram stool, tissue egg load and in total & female worm burden and oogram alteration.

We attributed these results to the inhibitory effect of artesunate on the juvenile stages of schistosomes in early stages of infection as well as its inhibitory effect on the reproductive system of both female and male adult worms in late infection. This opinion was in agreement with Xiao et al. (26) who attributed the reduction in egg count to the inhibitory effect of artemether on sexual maturation causing atrophy of testis and ovaries of the worms. In addition to, the reduction in egg load in artesunate-treated groups was explained by that artesunate modified the reproductive organs of *S. mansoni* female worms by reduction of ovarian volume and rarefaction of the vitel-line follicles (27). Moreover, residual worms recovered after artemether treatment became sterile and incapable of laying eggs (28). This observation approves that artesunate impairs the fecundity of adult female worms rather than affecting their count.

In the same context, PZQ monotherapy achieved cure rates ranged from 60% up to 90% but 100% cures were seldom if ever, recorded in an endemic area (4). PZQ had direct effect on adult schistosomes leading to their contraction, paralysis, and shift to the liver where they are finally destroyed by phagocytic cells (29). The schistosomes became most sensitive to PZQ 6w pi; the period of oviposition and plateau phase of parasite susceptibility (30)

It is preferred to use drugs acting on different stages of schistosome to achieve the radical cure (31). Since artesunate are more active against early developmental stages of schisto-some the time exactly when PZQ is ineffective so that treatment with PZQ in combination with any of artemisinin derivatives enables killing of both the schistosomules and mature adult worms. This is explained by hepatic shift of the worms, growth retardation and hence death of these worms.

Coming along, Elmorshedy et al. (32) recorded that following PZQ chemotherapy; the overall cure rate in children assessed by egg count in stool was 83.2%. While it was higher in Artemether-PZQ combination therapy being (87.2%), they also approved the prophylactic effect of artemether on human *Schistosoma mansoni* infection among Egyptian children.

Regarding the oogram alteration, the present results were similar to previous studies (8, 25). Furthermore, PZQ treatment 6w pi altered the oogram pattern and 97% of the ova were found dead (33).

Regarding the histopathological results, the combined treatment artesunate-PZQ exceeded PZQ monotherapy; also, artesunate monotherapy was nearly potent as the combined treated group regarding reduction in granuloma number & diameter, these results were in agreement of previous results (25, 33).

The combined artesunate-PZQ treated group 3w pi showed almost normal hepatic architecture with complete absence of granulomas this result was also recorded by several studies on artemether (13, 25). Artesunate was one of the artemisinin group as well as artemether, and both act as promising candidates for treatment and chemoprophylaxis of schistosomiasis. While the combined artesunate-PZQ treated group 6w pi was accompanied by fewer histopathological changes in the liver with marked reduction rates in granuloma number and diameter, atypical granulomas were seen with degenerated eggs.

Apoptosis is a tightly regulated physiological process in which cells establish an inducible cellular death of non-necrotic type. Apoptosis has a major role in balancing cell proliferation and remodeling tissue activities in many organisms (34). The present study was planned to study the hepatocyte induced apoptosis in experimentally infected mice with *S. mansoni* using immunohistochemical expression of P53 and Bcl-2 markers, to evaluate the therapeutic effect of PZQ, artesunate and their combinations in treatment of schistosomiasis *mansoni*.

In the current results, there was significant increase in the immunohistochemical changes and the incidence of apoptosis after *S. mansoni* infection. Immunohistochemical observations of the liver tissues showed a significant increase in the apoptotic proteins P53 and slight increase in anti-apoptotic proteins Bcl-2 in the infected group with *Schistosoma* cercaria compared with the control healthy group. A significant decrease and increase in the expression of P53 & Bcl-2 respectively were observed in artesunate - praziquantel combined group compared to control groups. Thus, the treatment with artesunate either alone or in combination with PZQ improved these apoptotic changes. This result was in accordance to Botros et al. (13). Besides, the control healthy group in this study was negative for both p53 and Bcl-2 markers expressions. This was in agreement with (12, 13).

In the present work, an opposite association or negative correlation was found between the expression of p53 and Bcl-2 proteins. Alike bidirectional changes of p53 and Bcl-2 expressions were also detected (24). Any decrease in apoptosis was associated with an increase in Bcl-2 expression in all studied groups (13). This was clarified by the fact that Bcl-2 expression (anti-apoptotic) was known to have protective effect against oxidative damage (35).

*S. mansoni* isolates with reduced susceptibilities to PZQ have already been identified (36). Schistosomiasis is distributed into new areas where climate change, especially temperatures, that become suitable for increased *S. mansoni* transmission over much of eastern Africa particularly in Burundi, Rwanda, eastern Zambia and south-west Kenya (37).

From our results we can gain insight into, artesunate- praziquantel combined group had the best results exceeded PZA monotherapy followed by artesunate monotherapy group regarding the reduction in stool egg count, tissue egg count (liver and intestine), worm burden, granuloma number & size and changed oogram patterns. In addition, Bcl-2 had an anti-apoptotic effect through its protective effect against oxidative damage, while P53 had apoptotic effect, and they were good markers for evaluation of *S. mansoni* morbidity and drug therapy.

## Conclusion

Artesunate-PZQ is a potential upcoming chemotherapy for schistosomiasis. Both Bcl-2 and P53 are good markers for assessment of *S. mansoni* apoptosis and chemotherapy efficacy. Artesunate and other artemisinin derivatives should be taken in human with caution, because of concern that this strategy might be selected for Plasmodium treatment and to avoid its resistant, such expectation is of debate by the malaria community.
